# Progressive Paralysis

**Published:** 1889-10-12

**Authors:** 


### SURGERY.

### PROGRESSIVE PARALYSIS.

A case of progressive paralysis of the musculo-spiral nerve consequent on injury, and cured by operation, has been recorded* by Mr. Chancy Puzey, surgeon to the Liverpool Northern Hospital. Three months after fracture of the middle of the humerus a man suffered from complete loss of power of extension of the hand and fingers, sensation being also defective. The muscles of the dorsum of the forearm were flabby and wasted, and supination of the forearm was difficult. The site of the fracture presented an excessive amount of callus. Pressure over, the course of the nerve elicited the ordinary sensations until that part over the callus was reached, when they ceased. It was, therefore, judged that the nerve was involved in the callus being pressed upon in some manner. An incision was made over the musculo-spiral groove, and the nerve exposed where it lay on the callus. On making a longitudinal incision through the neurilemma the nerve substance bulged out excessively, showing that it was much compressed. The nerve was dissected out for upwards of three inches. After about a week there slowly returned power of extension of the fingers and then of the wrist. Galvanism and massage were also applied, and three months afterwards the limb had completely recovered. Mr. Puzey observes that it may be objected that the operation had less to do with the result than the galvanism and massing. But he points out that up to the time of the operation the course was rapidly downhill, while after the operation the reverse obtained. It therefore seems almost certain that without surgical measures the man would have lost the use of his arm.

